# Coarctation of the aorta and persistent left superior vena cava: HDlive Flow features at 14 weeks of gestation

**DOI:** 10.1515/crpm-2021-0066

**Published:** 2022-02-01

**Authors:** Toshiyuki Hata, Aya Koyanagi, Riko Takayoshi, Takahito Miyake, Yuichiro Nakai, Kazumasa Tani, Kei Hayata, Hisashi Masuyama

**Affiliations:** Department of Obstetrics and Gynaecology, Miyake Clinic, Okayama, Japan; Department of Perinatology and Gynaecology, Kagawa University Graduate School of Medicine, Miki, Kagawa, Japan; Department of Obstetrics and Gynaecology, Kawasaki Medical School, Okayama, Japan; Department of Obstetrics and Gynaecology, Okayama University Graduate School of Medicine, Dentistry and Pharmaceutical Sciences, Okayama, Japan

**Keywords:** coarctation of aorta, early second trimester, HDlive Flow, persistent left superior vena cava, prenatal diagnosis, spatiotemporal image correlation (STIC)

## Abstract

**Objectives:**

A significant discrepancy between a large ductus arteriosus and a smaller aorta at their connection is key to diagnose coarctation of the aorta (CoA) at 14–16 weeks of gestation. CoA was associated with persistent left superior vena cava (PLSVC) in 21.3% of fetuses. HDlive Flow findings for CoA or PLSVC were obtained only in the third trimester of pregnancy. To the best of our knowledge, there has been no report on the prenatal findings of CoA and PLSVC using HDlive Flow with spatiotemporal image correlation (STIC) before 20 weeks of gestation.

**Case presentation:**

We present the trans-abdominal HDlive Flow features of CoA and PLSVC at 14 weeks of gestation. With a three-vessel trachea view on multiplanar view using color Doppler with STIC, PLSVC on the left side of the pulmonary artery was noted, and a narrowing aortic isthmus was suspected. A narrowing isthmus was also suspected with an aortic arch view. HDlive Flow clearly showed the spatial relationships among the right superior vena cava, aorta with narrowing isthmus, pulmonary artery, and PLSVC. A preductal ‘shelf’ was also suspected. No other fetal anomaly was noted. Neonatal echocardiography after delivery confirmed CoA and PLSVC.

**Conclusions:**

To the best of our knowledge, this is the first report on HDlive Flow features of fetal CoA and PLSVC using STIC early in the second trimester of pregnancy.

## Introduction

HDlive Flow with spatiotemporal image correlation (STIC) provides additional information for assessment of normal fetal cardiac anatomy and prenatal diagnosis of congenital heart disease (CHD) [1] because we can easily understand special relationships in normal and abnormal fetal cardiac structures. Moreover, even in the late first- and early second-trimesters, HDlive Flow with STIC is useful for the diagnosis of fetal CHD [2, 3]. In this investigation, we present HDlive Flow features of coarctation of the aorta (CoA) and persistent left superior vena cava (PLSVC) using STIC at 14 weeks of gestation.

## Case presentation

**Figure 1:**
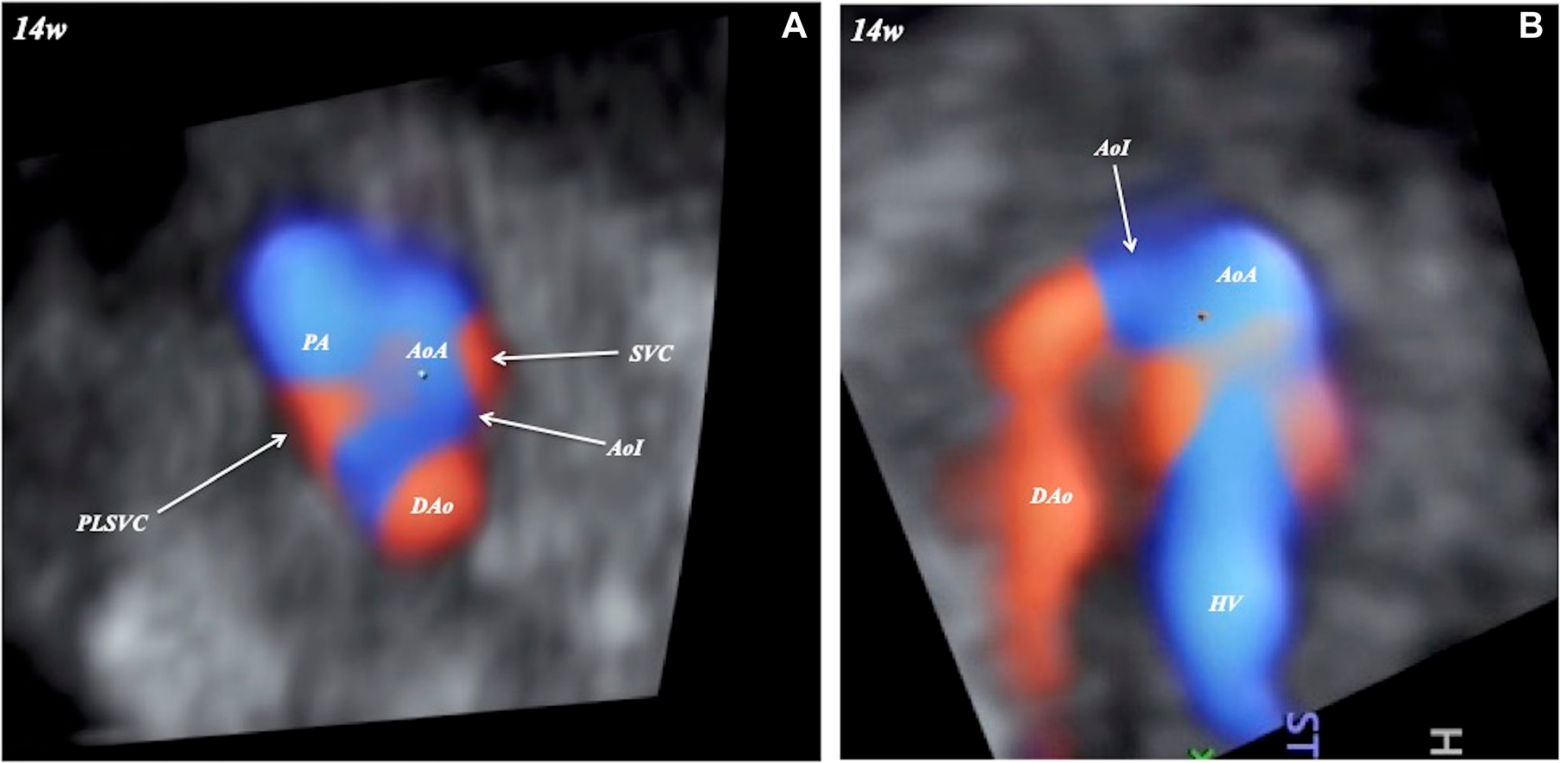
Multiplanar view with spatiotemporal image correlation of fetal coarctation of the aorta and persistent left superior vena cava (PLSVC) at 14 weeks of gestation.AoA, aortic arch; AoI, aortic isthmus; DAo, descending aorta; HV, hepatic vein; PA, pulmonary artery; SVC, superior vena cava. A, three-vessel trachea view; B, aortic arch view.

**Figure 2:**
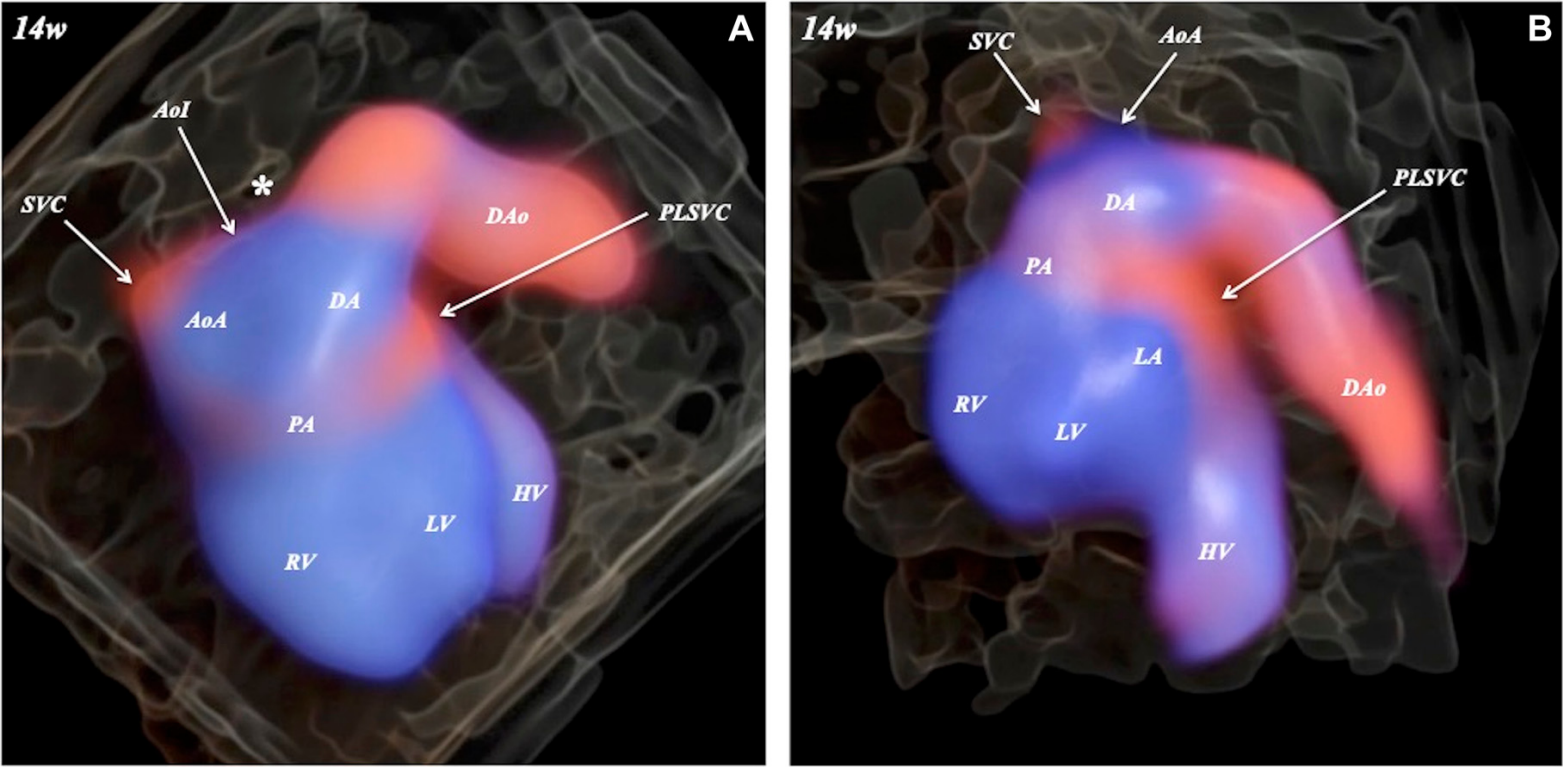
HDlive Flow image of fetal coarctation of the aorta and persistent left superior vena cava (PLSVC) at 14 weeks of gestation.Preductal ‘shelf’ (*) is suspected. AoA, aortic arch; AoI, aortic isthmus; DA, ductal arch; DAo, descending aorta; HV, hepatic vein; LA, left atrium; LV, left ventricle; PA, pulmonary artery; RV, right ventricle; SVC, superior vena cava. A, spatial three-vessel view; B, panoramic view.

A 33-year-old pregnant Japanese woman, G (1), P (0), received routine obstetrical screening at 14 weeks of gestation. Fetal biometry was consistent with the corresponding gestational age. With a three-vessel trachea view on multiplanar view by color Doppler with STIC, PLSVC on the left side of the pulmonary artery was noted, and a narrowing aortic isthmus was suspected (Figure 1A). A narrowing isthmus was also suspected with an aortic arch view (Figure 1B). HDlive Flow with STIC (Voluson E10 BT20, GE Healthcare, Zipf, Austria) with a curved array trans-abdominal transducer (GE eM6C G2, 2–7 MHz) clearly showed spatial relationships among the right superior vena cava, aorta with narrowing isthmus, pulmonary artery, and PLSVC (Figure 2A). A preductal ‘shelf’ was also suspected (Figure 2A). PLSVC could be clearly identified on the panoramic view (Figure 2B). No other fetal anomaly was noted. A narrowing isthmus and PLSVC with right ventricular dominance were confirmed in the second- and third-trimester scans. The pregnancy course was non-eventful.

A female newborn was vaginally delivered by vacuum extraction at 40 weeks and 6 days of gestation with a body weight of 2,586 g and length of 46 cm. The umbilical artery pH was 7.288. She had an Apgar score of 8/9 at 1 and 5 min, respectively. Neonatal echocardiographic diagnosis was CoA (isthmus diameter=2.4 mm) (normal range of isthmus diameter for Japanese infant with a height of 46 cm: 2.51–8.79 mm) [4] and PLSVC. No blood pressure difference was noted between the arm and leg. There was no need for prostaglandin E1 use after delivery. On the 28th neonatal day, ductus arteriosus closure was confirmed with color Doppler ultrasound, and no effect on the circulatory hemodynamics of the baby was ascertained. So, close follow-up was decided. The neonate followed a favorable course without operation after delivery.

## Discussion

Transvaginal two-dimensional sonography was useful to suspect CoA at 14–16 weeks of gestation [5]. CoA could also be detectable at 11–13 + 6 weeks of gestation [6], and narrowing isthmus was the direct finding of CoA in the late first and early second trimesters of pregnancy [7]. The earliest prenatal diagnosis of PLSVC was at 16 weeks of gestation [8]. An association with CoA was noted in about one fifth of cases with PLSVC [9]. Three-vessel trachea view is the most important diagnostic clue to detect a narrowing isthmus and a blood vessel on the left side of the pulmonary artery for the prenatal diagnosis of CoA and PLSVC [10]. STIC is reliable not only for early reassurance of normal cardiac anatomy but also to diagnose CHD before 16 weeks’ gestation [11]. In the present investigation, a multiplanar view with STIC at 14 weeks of gestation clearly showed PLSVC on the left side of the pulmonary artery and led to a suspected narrowing isthmus on the three-vessel trachea view. Moreover, HDlive Flow clearly demonstrated spatial relationships among the right superior vena cava, aorta with narrowing isthmus, pulmonary artery, and PLSVC, and also suggested a preductal ‘shelf’. To the best of our knowledge, this is the first report on HDlive Flow features of fetal CoA and PLSVC using STIC early in the second trimester of pregnancy. HDlive Flow with STIC may provide additional information to diagnose CHD before 15 weeks’ gestation.

## References

[1] Ito M, AboEllail MAM, Yamamoto K, Kanenishi K, Tanaka H, Masaoka H (2017). HDlive Flow silhouette mode and spatiotemporal image correlation for diagnosing congenital heart disease. Ultrasound Obstet Gynecol.

[2] AboEllail MAM, Kanenishi K, Tenkumo C, Kawanishi K, Kaji T, Hata T (2015). Diagnosis of truncus arteriosus in first trimester of pregnancy using transvaginal four-dimensional color Doppler ultrasound. Ultrasound Obstet Gynecol.

[3] Hata T, Ito M, Nitta E, Pooh R, Sasahara J, Inamura N (2018). HDlive Flow silhouette mode for diagnosis of ectopia cordis with a left ventricular diverticulum at 15 weeks’ gestation. J Ultrasound Med.

[4] Ikado H, Tsukano S, Itoh S, Kohyama K, Tanaka N, Nakazone I (2010). Normal value of aortic size and morphological characteristics of the aorta in childhood evaluated by two-dimensional ultrasonography. Ped Cardiol Card Surg.

[5] Bronstein M, Zimmer EZ (1998). Sonographic diagnosis of fetal coarctation of the aorta at 14–16 weeks of gestation. Ultrasound Obstet Gynecol.

[6] Hernamdez-Andrade E, Patwardhan M, Cruz-Lemini M, Luewan S (2017). Early evaluation of the fetal heart. Fetal Diagn Ther.

[7] Carvalho JS, Moscoso G, Tekay A, Campbell S, Thilaganathan B, Shinebourne EA (2004). Clinical impact of first and early second trimester fetal echocardiography on high risk pregnancies. Heart.

[8] Choi EY, Hong SK, Jeong NY (2016). Clinical characteristics of prenatally diagnosed persistent left superior vena cava in low-risk pregnancies. Prenat Diagn.

[9] Gustapane S, Leombroni M, Khalil A, Giacci F, Marrone L, Bascietto F (2016). Systematic review and meta-analysis of persistent left superior vena cava on prenatal ultrasound: associated anomalies, diagnostic accuracy and postnatal outcome. Ultrasound Obstet Gynecol.

[10] Wen TM, Huang YL, Wu PC, Li YY, Chen MR, Chang TY (2017). Prenatal diagnosis of persistent left superior vena cava is associated with coarctation of the aorta – a case report. J Med Ultrason.

[11] Bennasar M, Martinez JM, Olivella A, Del Rio M, Gomez O, Figueras F (2009). Feasibility and accuracy of fetal echocardiography using four-dimensional spatiotemporal image correlation technology before 16 weeks’ gestation. Ultrasound Obstet Gynecol.

